# The role of comprehensive rehabilitation in the care of degenerative cervical myelopathy

**DOI:** 10.1038/s41393-024-00965-y

**Published:** 2024-03-04

**Authors:** Amiram Catz, Yaron Watts, Hagay Amir, Lilach Front, Ilana Gelernter, Dianne Michaeli, Vadim Bluvshtein, Elena Aidinoff

**Affiliations:** 1 The Spinal Rehabilitation Department, Loewenstein Rehabilitation Medical Center, Raanana, Israel; 2 The Rehabilitation Department, Tel-Aviv University, Tel-Aviv, Israel; 3 The Orthopedic Rehabilitation Department, Loewenstein Rehabilitation Medical Center, Raanana, Israel; 4 The Statistical Laboratory, School of Mathematics, Tel-Aviv University, Tel-Aviv, Israel; 5 The Intensive Care for Consciousness Rehabilitation Department, Loewenstein Rehabilitation Medical Center, Raanana, Israel

**Keywords:** Spinal cord diseases, Rehabilitation, Outcomes research

## Abstract

**Study design:**

Retrospective cohort study.

**Objective:**

To find out if comprehensive rehabilitation itself can improve daily performance in persons with DCM.

**Setting:**

The spinal department of a rehabilitation hospital.

**Methods:**

Data from 116 DCM inpatients who underwent comprehensive rehabilitation after spinal surgery were retrospectively analyzed. The definitions of the calculated outcome variables made possible analyses that distinguished the effect of rehabilitation from that of spinal surgery. Paired *t*-tests were used to compare admission with discharge outcomes and functional gains. Spearman’s correlations were used to assess relationships between performance gain during rehabilitation and between time from surgery to rehabilitation.

**Results:**

The Spinal Cord Injury Ability Realization Measurement Index (SCI-ARMI) increased during rehabilitation from 57 (24) to 78 (19) (*p* < 0.001). The Spinal Cord Independence Measure 3^rd^ version (SCIM III) gain attributed to neurological improvement (dSCIM-IIIn) was 6.3 (9.2), and that attributed to rehabilitation (dSCIM-IIIr) 16 (18.5) (*p* < 0.001). dSCIM-IIIr showed a rather weak negative correlation with time from spinal surgery to rehabilitation (*r* = −0.42, *p* < 0.001).

**Conclusions:**

The study showed, for the first time, that comprehensive rehabilitation can achieve considerable functional improvement for persons with DCM of any degree, beyond that of spinal surgery. Combined with previously published evidence, this indicates that comprehensive rehabilitation can be considered for persons with DCM of any functional degree, before surgery.

## Introduction

Degenerative cervical myelopathy (DCM) is a common non-traumatic spinal cord disorder [1]. Publications on DCM estimated that non-operative care led to deterioration in functional status in 20–62% of individuals with DCM within 3–6 years of follow-up, whereas DCM surgery was described as safe and effective [1]. Consequently, the literature considers DCM a progressive neurological condition that requires early referral for evaluation of surgical decompression to prevent poor outcomes [2]. Some support early surgical intervention in persons with DCM of any severity [3]. The DCM literature inferred on the need for surgical interventions, based on reports on non-operative interventions that did not prevent functional deterioration [1]. These interventions included the use of medication, such as anti-inflammatory drugs, muscle relaxants, gabapentin and pregabalin, transforaminal or epidural spinal injections, intermittent bed rest, cervical immobilization, cervical traction, manipulation therapy, thermal therapy, discouragement of high-risk activities, avoidance of risky environments, not-specified physical therapy, not-specified home exercise, and sporting activities [4, 5].

These interventions, however, may not adequately represent the conservative care that can be offered to individuals with DCM. Each of them addresses only some of the factors that influence the performance of persons with DCM. Yet, the DCM literature has not considered comprehensive rehabilitation, as practiced in spinal cord lesions (SCL) units, as a substantive option for the treatment of DCM of any degree, although it addresses a set of factors that affect performance, from various domains, and demonstrated good results in people after traumatic and non-traumatic SCL [6, 7]. Although ignored in the DCM literature, such comprehensive rehabilitation is customary for persons with DCM, which is a type of SCL, as it is for other individuals with SCL, mainly after spine surgery [1, 7].

Comprehensive rehabilitation is carried out in SCL units by a multidisciplinary team managed by physiatrists [6, 8]. These rehabilitation teams usually include nurses, physiotherapists, occupational therapists, social workers, and psychologists; they regularly consult spinal surgeons, urologists, psychiatrists, plastic surgeons, otolaryngology specialists, and speech therapists [6].

Comprehensive SCL rehabilitation has two main objectives that are highly relevant for persons with neurological and functional deficits caused by DCM: (a) preventing medical complications of SCL (pneumonia, pressure sores, urinary infections, vascular autonomic impairments, and others) to prolong life and avoid loss of function; and (b) minimizing the gap between the potential and actual functioning of each person, aimed at minimizing complications and improving function and quality of life [8].

Potential functioning (or potential performance) is determined by the damage to neural tissue and by neurological status [3, 8, 9]. Functioning and disability after SCL may be affected by various factors, including the neurological status reflected in SCL severity and level, the time from injury to examination, the realization of the patient’s potential performance before rehabilitation, and to some extent, the length of stay in rehabilitation, age, presence of pressure sores or spasticity, pain, psychological factors, and environmental factors [8, 10–12]. Of all these, spine surgery can affect only the neurological status and to some extent pain. Spine surgery, therefore, can reduce disability mainly by improving potential performance following the improvement of the neurological status. By contrast, comprehensive rehabilitation can reduce disability mainly by increasing the realization of potential performance, although it may affect the assessment of neurological status, to some extent, by training, which strengthens the muscles [8, 9]. In other words, comprehensive rehabilitation can increase ability realization, which is quantitatively described as the ratio of the values of actual performance and potential performance (Table 1) [8, 9]. It increases ability realization by preventing and treating SCL complications, reducing spasticity and pain, improving mood and motivation, and delivering education and training, which may harness the plasticity of the central nervous system [8, 13].

**Table 1:** Measures used in the present study and in the DCM literature.

Measure	Description
AMS	American Spinal Injury Association motor score. It is included in the international standards for neurological classification of spinal cord injury, and represented the persons’ neurological motor status (score range 0-100) [14].
SCIM-III	Spinal Cord Independence Measure third version (SCIM-III). It is a comprehensive measure of actual daily task performance, designed specifically for persons with spinal cord lesions. It was internationally validated, and is used today in spinal cord units worldwide (score range 0-100) [15].
SCIM95	The 95th percentile of SCIM III value, representing the potential performance for each AMS. It is calculated using a formula that includes AMS and SCIM-III values, and controls for age (years) and gender (Male = 0, Female = 1). It was found stable and valid for several countries: SCIM95 = 26.017 − [0.004 × (AMS^2^)] + [1.236 × AMS] − [0.127 × Age] − 3.674 × Gender [9].
SCI-ARMI	Ability realization as a %. It is a measure of the realization of the potential performance, which equals SCIM-III/SCIM95×100 [9].
dSCIM-III	Value of the improvement in actual performance during comprehensive rehabilitation, which is the difference between SCIM III values at discharge and admission, or SCIM-III gain, which equals (SCIM-III_2_)–(SCIM-III_1_).
dSCIM-IIIn	The portion of SCIM-III gain that was due to neurological motor change during rehabilitation alone. It is obtained by calculating the difference between discharge and admission potential performance (SCIM-III95_2_, and SCIM-III95_1_), which is 100(SCIM-III_2_/SCIARMI_2_–SCIM-III_1_/SCIARMI_1_).
dSCIM-IIIr	The portion of SCIM-III gain that was due to the change in ability realization and not to neurological motor change during rehabilitation. It is obtained by calculating the difference between dSCIM-III and dSCIM-IIIn, which is SCIM-III_2_–SCIM-III_1_-100(SCIM-III_2_/SCIARMI_2_–SCIM-III_1_/SCIARMI_1_.
dSCI-ARMI	The change in ability realization. It is obtained by calculating the difference in SCI-ARMI values between discharge and admission.
JOA, mJOA Nurick, NDI	JOA or mJOA scores, the Nurick grades, and the Neck Disability Index (NDI) are tests that primarily asses activities of daily living [3, 16, 19–25, 28].
Timed 10-MWT	Timed 10-Meter Walk Test is a measures for gait assessment [28].
Quick DASH	Quick DASH is a measure of upper limb function [16].
OFSR, RTW rate	Overall Functional Status Rating and the Return to work rate are measures of participation in social and vocational activities [28, 29].

Although comprehensive rehabilitation was effective for persons with SCL, we found no studies that examined it separately for an SCL subgroup with DCM [6, 7]. We conducted the present study to assess the contribution of comprehensive rehabilitation itself to improving disability in this subgroup and to evaluate its role in DCM care.

## Methods

### Study population

Persons admitted consecutively to the spinal department of a rehabilitation medical center, between 2011 and 2020, were enrolled in this retrospective cohort study. Inclusion criteria were non-traumatic complete or incomplete tetraplegia, mild to severe disability, and degenerative changes of the cervical spine. Included were only individuals with spinal surgery for degenerative cervical spine changes conducted before admission because the majority of the individuals with DCM admitted to the spinal department during the study period were referred after a spinal operation. Exclusion criteria were additional medical conditions assessed as influencing the neurological status or the performance, such as additional spinal lesions, brain or limb injury, acute illness, or missing relevant data.

### The collected and calculated data

The authors retrospectively collected patient demographic and clinical data and SCL characteristics from the hospital records of the patients. The collected data of the American Spinal Injury Association motor score (AMS, score range 0–100), and the Spinal Cord Independence Measure (SCIM) third version (SCIM-III, score range 0–100), represented the persons’ neurological motor status and performance, respectively (Table 1) [14, 15]. The collected AMS and SCIM III data were from the first week after admission to inpatient rehabilitation and the week before discharge.

### Data analysis

Analyses distinguished the effect of rehabilitation from that of spinal surgery on patient performance. We used SCIM III and AMS scores to calculate separate variables for the assessment of the contribution of rehabilitation and neurological change to the change in performance (dSCIM-IIIr and dSCIM-IIIn), and the values of the Spinal Cord Injury Ability Realization Measurement Index (SCI-ARMI) (Table 1).

We used paired two-tailed t-tests to compare admission and discharge outcomes, and dSCIM-IIIr and dSCIM-IIIn. We used Spearman’s correlation test to assess the relationship between the gain in performance during rehabilitation and the time from spinal surgery to rehabilitation. We compared the persons included in the study with those excluded from it using independent sample two-tailed t-tests for continuous variables, and chi-square or Fisher’s exact tests for categorical ones. All the statistical tests were chosen after examination of the distribution, variability, or the ordinality of the variables’ data. Statistical analyses were performed using the Statistical Package for the Social Sciences (SPSS version 27.0, Chicago, IL, USA).

### Ethical considerations

The institutional review board (IRB) of the rehabilitation medical center approved the study. The investigators followed the ethical principles of the Declaration of Helsinki. The requirement for informed consent from the study subjects was waived by the IRB due to the retrospective study design.

## Results

### Patients

Of 220 eligible patients, 104 met exclusion criteria, leaving a sample of 116 individuals. The indication for surgery in all these patients was a diagnosis of cervical spinal stenosis and/or disc protrusion, usually with corresponding neurological findings, and/or MRI findings compatible with cervical myelopathy. The patients underwent a rehabilitation program carried out by a multidisciplinary team and managed by physicians specializing in rehabilitation medicine and the care of SCL. The program was standardized by setting goals that represent achievements assessed as the maximum possible for each patient. It included (a) medical and nursing measures, used in consultation with various specialists, to prevent and treat medical complications of SCL; (b) training by the nursing, physiotherapy, and occupational therapy staff, using manual and advanced technological techniques to improve strength, ranges of motion, and functioning, aiming for the potential functioning that can be achieved with the neurological status the patients reached; and (c) evaluation, support, consultation, and interventions, by psychologists and social workers to assist in coping with disability and negative reactions, and help to settle and live in the community after discharge. The program involved periodic monitoring of neurological and function statuses and of computed SCI-ARMI values, which assisted in the assessment of progress, revealing obstacles in the way to achieving maximum performance, and taking measures to improve it.

The length of stay in rehabilitation (LOS) was 72 days (SD = 40, Table 2), and patients were usually discharged when their medical condition stabilized and allowed community care, and their functional status reached a plateau.

**Table 2:** Patient characteristics.

Measure	DCM patient group			
	N/Mean	SD/%	Median	Range
Age (years)	60.6	12.0	60.8	23–85
Male gender	85	73.3%		
AIS grade A	0	0%		
AIS grade B	2	1.7%		
AIS grade C	14	12.1%		
AIS grade D	100	86.2		
Level C1-3	25	21.6%		
Level C4	63	54.3%		
Level C5	19	16.4%		
Level C6	4	3.4%		
Level C7-8	5	4.3%		
T onset (days)	516	963	187	6–7264
T surg (days)	172	779	17	4–7264
LOS (days)	72	40	65	12–196

*Level* the level of the lowest intact spinal cord segment, *N* the number of patients in each group, *SD* standard deviation, *T onset* time from lesion onset to admission to comprehensive rehabilitation, *T surg* time from spinal operation to admission to comprehensive rehabilitation, *LOS* length of stay in comprehensive rehabilitation.

Comparison of characteristics of the included individuals (Table 2) and those excluded shows no statistically significant differences in age, gender, LOS, admission SCIM-III and SCI-ARMI scores, and admission AIS grade (*p* > 0.05). Admission AMS was 74.9 (SD = 19.8) for the persons included in the study, and 67.8 (SD = 19.0) for those who were excluded (*p* < 0.01). The calculated values of neurological and performance measures are shown in Table 3.

**Table 3:** Observed and calculated values of neurological and performance measures.

Measure	DCM patient group			
	Mean	Median	SD	Range
AMS-1	74.9	81.0	19.8	4–100
AMS-2	85.0	87.5	15.3	16–100
SCIM-III-1	49.5	50.0	23.4	4–100
SCIM-III-2	71.5	77.0	20.5	10–100
dSCIM-III	22.0	18.0	17.8	(−13)–68
SCIM95-1	84.3	89.7	15.0	17–103
SCIM95-2	90.5	93.4	10.8	34–103
SCI-ARMI-1	57.3	58.0	24.1	12–110
SCI-ARMI-2	77.9	82.5	19.2	26–112
dSCI-ARMI	20.5	16.9	19.4	(−20)–67
dSCIM-IIIn	6.3	3.6	9.2	(−9)–51
dSCIM-IIIr	15.8	12.5	18.5	(−24)–65

*SD* standard deviation, *AMS* American Spinal Injury Association Motor Score, 1 = at admission to rehabilitation, 2 = at discharge from rehabilitation, *d* delta, *SCIM-III* Spinal Cord Independence Measure third version, *dSCIM-III* SCIM-III_2_ - SCIM-III_1_, *SCI-ARMI* Spinal Cord Ability Realization Measurement Index, *dSCI-ARMI* SCI-ARMI_2_ - SCI-ARMI_1_, *dSCIM-IIIn* 100(SCIM-III_2_/SCIARMI_2_ –SCIM-III_1_/SCIARMI_1_), dSCIM-IIIr dSCIM-III- dSCIM-IIIn.

### Outcomes of comprehensive rehabilitation

The persons with DCM included in this analysis improved in neurological motor status and performance (AMS gain = 10.1, SD = 12.7, *p* < 0.001; SCIM III gain=22.0, SD = 17.8, *p* < 0.001). They also improved in ability realization (dSCI-ARMI = 20.5 SD = 19.4, *p* < 0.001). Of the improvement in performance, 71% (dSCIM-IIIr=16) can be attributed to improved ability realization and 29% (dSCIM-IIIn=6) to neurological motor improvement (*p* < 0.001).

**Fig. 1:**
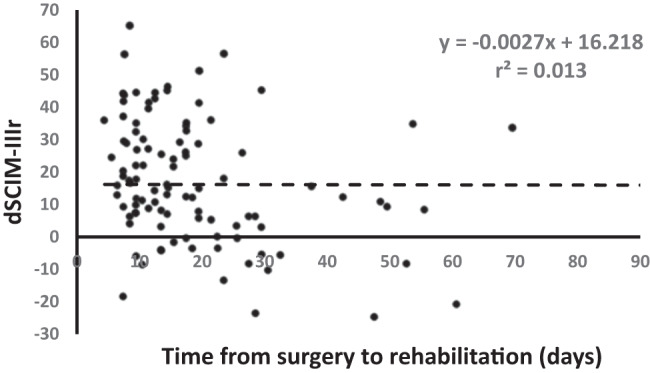
Relationship between the portion of performance gain during rehabilitation, which was independent of neurological improvement during rehabilitation, and the time from spinal surgery to admission to rehabilitation. The majority of the patients entered into the study were within 70 days of spinal surgery. For these, the time from surgery to rehabilitation did not affect the portion of the gain in performance during rehabilitation, which was independent of the neurological improvement during rehabilitation.

The functional gain, dSCIM-III, and the functional gain not attributed to motor neurological improvement, dSCIM-IIIr, showed a rather weak negative correlation with the time from spinal surgery to rehabilitation (*r* = −0.399, and −0.416, *p* < 0.001, Fig. 1). The correlation between dSCIM-IIIn and the time from spinal surgery to rehabilitation was not significant.

## Discussion

### Comprehensive DCM rehabilitation outcomes

The present study showed, for the first time, that comprehensive rehabilitation, as practiced in SCL units, administered by a multidisciplinary team, and implementing combined medical and para-medical specific skills to prevent medical complications of SCL, and improve patient functioning, achieved considerable functional gain for persons with DCM of all functional levels, beyond the gain that spinal surgery alone achieved. To assess the net effect of rehabilitation, it would have been reasonable to examine DCM individuals who received only rehabilitation or compare our patients with DCM patients who underwent spinal surgery without rehabilitation. This was not feasible in a retrospective study because (a) the prevailing approach advocates early referral of persons with DCM to surgical decompression, which limited the referral of persons with DCM to rehabilitation before spinal surgery, and (b) the assessments used in this study were not available for patients who were not admitted to rehabilitation. Nevertheless, we inferred about the contribution of rehabilitation alone to improved functioning. Our analyses distinguished the effect of rehabilitation from that of spinal surgery, isolating the effect of late post-surgical motor neurological change on daily performance during rehabilitation and examining the relationship of the functional gain during rehabilitation with the time from spinal surgery to rehabilitation.

In the study group, motor neurological scores increased during rehabilitation by 13%, daily functioning scores by 44%, and scores for ability realization by 37%. The analysis indicated that less than 30% of the improvement in the performance of daily activities during rehabilitation can be attributed to motor neurological recovery that may be related to the late effect of surgery, and about 70% to the increase in ability realization.

It may be argued that a substantial part of the improvement in performance during rehabilitation, which was not related to neurological changes that occurred during the same period, was a late response to changes caused by the surgery, before the start of rehabilitation. A possible interpretation of this claim is that rehabilitation hastens the rate of gain in performance, but the functional outcome of patients who do not undergo rehabilitation is improving irrespective of the neurological change during rehabilitation, and may ultimately be the same as of those undergoing rehabilitation without concomitant neurological improvement, even if it at a slower rate and over a longer period. We expect, however, that the influence of substantial changes related to surgery, which occurred before the rehabilitation, and are not related to the neurological improvement during rehabilitation, would substantially decrease with the time after the operation, before and during the rehabilitation. Thus, we expect that dSCIM-IIIr would also substantially decrease with the time after the operation. But the correlation between dSCIM-IIIr and the time from spinal surgery to rehabilitation was rather weak, as demonstrated in Fig. 1, which suggests that the contribution of surgical effects to the functional gain during rehabilitation, not related to the neurological recovery during rehabilitation, was minor. This supports the attribution of dSCIM-IIIr mainly, even if not only, to the comprehensive rehabilitation. We can safely extrapolate, therefore, that in patients with DCM, a considerable improvement in performance can be attained by rehabilitation alone, irrespective of the contribution of surgery to performance. Consequently, comprehensive rehabilitation can be offered as adequate conservative care for improving functioning in persons with DCM of all functional degrees.

### Considering comprehensive rehabilitation before surgery for DCM

Although comprehensive rehabilitation achieved considerable functional gain for persons with DCM, beyond that of spinal surgery alone, based on the customary approach described in the Introduction, it should be offered only after spinal surgical intervention, at least for individuals with moderate or severe myelopathy [1, 3]. This approach is based on the notion that surgical intervention is more effective and safer than conservative care for these persons. But based on our findings, combined with published data of other studies, comprehensive rehabilitation can be at least as effective, and even safer, as shown below, and therefore should be tried in many cases, before the decision on surgery.

Careful assessment of the DCM literature reveals that the customary approach is not the unequivocal conclusion offered by the published data, because (a) a significant portion of the persons with DCM is at relatively low risk for deterioration with conservative care, contrary to the prevailing notion; (b) in patients with mild, moderate, or severe DCM, the estimated risk of deterioration and complications in the DCM literature is not necessarily lower after surgery than with conservative care; and (c) the deterioration rate under conservative care and the role of neurological change in inducing deterioration may be overestimated in the literature. We address these three points below.

- The prevailing notion that the risk of deterioration in persons with DCM under conservative care is very high was adopted, based on an estimated deterioration rate of 20–62% [1]. Based on the published data, however, it is more plausible to conclude that the risk of deterioration in persons with DCM under conservative care is not high because this estimated deterioration rate indicates that 38–80% of the persons with DCM do not suffer from functional deterioration within 3–6 years of follow-up, and in those with mild DCM, more than 50% can remain unchanged or improve [1, 16].
- If appropriate comparisons are used, the relative risk of deterioration and complications may be lower with conservative care than after surgery. The notion that surgery is safer for individuals with DCM is based on comparisons of the risk of deterioration after surgery with the total risk of deterioration with conservative care [1, 3]. But deterioration is attributed to surgery only if it occurs soon thereafter. Its risk should be compared with the risk of rapid deterioration that occurs soon after conservative care, combined with neurological follow-up. A reasonable estimation of the risk of rapid deterioration (with a course of less than 1–3 months) is between 1.4–13%, and near 7% on average (Table 4) [16–19]. A reasonable estimation of the average risk of all types of neurological deterioration, peri-operatively or early after DCM surgery is near 9% on average (Table 4) [5, 20–24]. Furthermore, Nakashima and colleagues described late neurological deterioration in 14% of people after laminoplasty, overall, 8.9–30.6% of patients suffered from at least one complication of surgery, and mortality was noted following up to 2.1% of spinal operations for DCM [5, 20–27].

**Table 4:** Published risks of DCM care.

Type of risk	Type of care	Course or time to event	DCM degree	Risk in %	References
Rapid deterioration	Conservative	Course of <3 months	Any	1.4–6.6	16 (2021)
Rapid deterioration	Conservative	Course <1 month	Moderate or severe	4–13	17 (2021)
Rapid deterioration	Conservative	Course <1 month (50% due to trauma)	Moderate or severe	18.6 (8 of 43)	18 (2015)
Traumatic event	Conservative	44 months follow-up	Asymptomatic	7	19 (2011)
Nerve root injury	Surgical	<3 months from surgery	Any	0.4–2.3	20 (2023)
Nerve root injury	Surgical	<3 months from surgery	Any	3.1–9.1	21 (2020)
Nerve root injury	Surgical	<3 months from surgery	Any	2.59	23 (2019)
Nerve root injury	Surgical	<3 months from surgery	Any	0–30	5 (2018)
Myelopathy pr/SCI	Surgical	<3 months from surgery	Any	0.8–3.5	20
Myelopathy pr/SCI	Surgical	<3 months from surgery	Any	2	22
Myelopathy pr/SCI	Surgical	<3 months from surgery	Any	6.74	23
Myelopathy pr/SCI	Surgical	<3 months from surgery	Any	0.01–0.3	5
Limb par/weak	Surgical	<3 months from surgery	Any	2.4–9.9	20
Limb par/weak	Surgical	<3 months from surgery	Any	13.3	20 (2021)
LNP	Surgical	<3 months from surgery	Any	3–4	5
LNP	Surgical	<3 months from surgery	Any	0–6.3	21
LNP	Surgical	<3 months from surgery	Any	6.9	24
Dysphagia	Surgical	<3 months from surgery	Any	0–16	21–24, 25 (2021)
Infection	Surgical	<3 months from surgery	Any	2.1–10.2	20,22–25, 27 (2022)
Other medical complications	Surgical	<3 months from surgery	Any	0.5–8	20,22–25,27

On average, after DCM surgery, nerve root injuries were reported in about 6%, worsening or progression of myelopathy or spinal cord injury 3%, limb paralysis or weakness 10%, laryngeal nerve palsy 4.5%, dysphagia 9.28%, and infection 6.42%. These operations had additional minor operative and hardware complications. Overall, 8.9%-30.6% of patients suffered from at least one complication of surgery. The risk values are those in the references, or calculated using their data.

*Myelopathy* pr/SCI Myelopathy progression or spinal cord injury, *Limb par/weak* limb paralysis or weakness, *LNP* laryngeal nerve palsy.
- The deterioration rate under conservative care and the role of neurological change in inducing it may be overestimated because most DCM studies based the assessment of clinical deterioration mainly on reports of performance. Despite claims of having assessed neurological change, these studies offer limited neurological information (Table 1) [3, 16, 19–25, 28, 29]. Assessments of performance, including those based on the JOA and mJOA scores, and subjective reports may be affected by several factors modifiable by medication and rehabilitation, and are not necessarily related to the DCM or the surgical intervention [1, 22]. Among these factors are pain, spasticity, motor skills in the presence of abnormal neurological status, fitness, mood, motivation, and primary or secondary gain. The effects of such factors may bias the assessment of change in performance [9]. Quantitative measures of the neurological status revealed a gap between neurological and functional change: Morishita and colleagues described chronic DCM patients with AMS of 91.8 ± 6.4, which represents a relatively mild neurological impairment, but with JOA scores of 10.1 ± 1.8, which represents severe disability. They demonstrated a degree of disability that exceeds that expected based on the DCM-related neurological deficit [18].

These insights suggest that repeated assessments can identify most of the individuals who deteriorate with conservative care before a significant functional change occurs, provided the assessments are sufficiently responsive to neurological changes. Based on the cited literature data (Table 4, if) these individuals are identified during the follow-up and undergo a decompressive operation only when deterioration starts, the overall neurological deterioration and risk of surgical complications for the entire DCM population will most likely be lower than the overall risk with the current customary indications for DCM surgery. Persons with DCM of all grades, who do not show deterioration in repeated neurological assessments, may therefore safely choose conservative care with neurological follow-up, avoiding operative complications for at least several years. For many persons with DCM of all grades, such conservative care can plausibly start with comprehensive rehabilitation, and surgical intervention may be considered if deterioration starts or comprehensive rehabilitation fails to improve performance.

### Considering modifications in the guidelines for DCM follow-up and care

Based on our findings, which support the advantage of comprehensive rehabilitation, and on literature data challenging the extent of spine surgery benefits for persons with DCM, we suggest considering the introduction of the following changes into guidelines for DCM follow-up and care:

1. **Introduction of quantitative neurological assessment**. For the detection of improvement or deterioration in persons with DCM, a physician or an experienced caregiver should use AMS, ASS and proprioception assessment [14].
2. **Introduction of quantitative assessment of performance**. For the assessment and follow-up of the performance of daily activities, an experienced caregiver should use SCIM III [11]. SCI-ARMI can be used to assess the potential to improve performance in rehabilitation [9].
3. **Changing recommendations for interventions**. Customary guidelines recommending surgical intervention for severe and moderate DCM, and offering a choice of surgical intervention or a supervised trial of structured rehabilitation for mild DCM, should be changed [1, 2] as follows: (1) Individuals with DCM and none or minimal and stable neurological and functional deficit should be followed up. Those with disruptive minimal deficits should be referred to ambulatory physical or occupational therapy. (2) Individuals with DCM with more than minimal and stable neurological or functional deficit should be assessed in a spinal rehabilitation facility for quantitative neurological status, actual performance, and the potential to improve performance in rehabilitation. Based on this assessment, patients should be referred to ambulatory rehabilitation, inpatient comprehensive rehabilitation, or consultation with a spine surgeon. (3) Surgical intervention should be considered if deterioration is evident in quantitative neurological assessments. Surgery may be also considered if actual performance is significantly impaired and close to potential performance, which means that the disability is significant and the potential to improve it in rehabilitation is poor.
4. **Introduction of follow-up instructions**. Regular follow-up should follow ambulatory or inpatient rehabilitation. When DCM is diagnosed or clinical worsening is reported, initial follow-up should be conducted weekly, to enable identification of deteriorations that may occur within less than two weeks. If no deterioration is detected after two weeks, the follow-up should be monthly, to enable identification of deterioration that may occur within 1 month. After 3 months, it should be repeated every 3 months, to enable identification of deterioration that may occur within 3 months, and after a year without deterioration, every year, to enable identification of late deterioration.
5. **Introduction of instructions for post-operative assessment and care**. After surgical intervention for DCM, patients should be referred to assessment in a SCL rehabilitation unit. Based on the findings of this assessment, patients should be referred to follow-up, ambulatory rehabilitation, or comprehensive inpatient rehabilitation.

### Limitation of the study

A limitation of the present study is the lack of patients who received only surgical or only non-surgical treatment in the study sample. Our analyses circumvented this consequence of the prevailing approach to treating DCM.

### Future research

To support our findings and their generalizability to all individuals with DCM, we recommend performing a study comparing patient groups after comprehensive rehabilitation alone and after surgery alone. To enable such a comparison, however, the guidelines for DCM care should be modified, as suggested in this article, at least for participants of the comparative study.

## Conclusions

Comprehensive rehabilitation can significantly improve daily performance in persons with DCM, beyond the improvement achieved by spinal surgery, and irrespective of its contribution. Based on this understanding and on literature data challenging the extent of spine surgery benefits for persons with DCM, we suggest considering modifying the recommendations for DCM follow-up and care, as detailed above. This would contribute to reducing the overall disability of individuals with DCM, improve the validity of assessing DCM clinical severity, and allow a direct comparison of the effects of rehabilitation and surgery.

### Reporting summary

Further information on research design is available in the Nature Research Reporting Summary linked to this article.

### Supplementary information

Reporting summary

## Data Availability

The data presented in this study are available on request from the corresponding author.
